# A Novel Defined Pyroptosis-Related Gene Signature for the Prognosis of Acute Myeloid Leukemia

**DOI:** 10.3390/genes13122281

**Published:** 2022-12-03

**Authors:** Kecheng Huang, Linka Xie, Fan Wang

**Affiliations:** 1 Department of Obstetrics and Gynecology, Tongji Hospital, Tongji Medical College, Huazhong University of Science and Technology, Wuhan 430030, China; 2 Cancer Center, Union Hospital, Tongji Medical College, Huazhong University of Science and Technology, Wuhan 430030, China; 3 Department of Hematology, Tongji Hospital, Tongji Medical College, Huazhong University of Science and Technology, Wuhan 430030, China

**Keywords:** pyroptosis, AML, prognosis, Tregs, TME, immune evasion

## Abstract

Pyroptosis is an inflammatory form of programmed necrotic cell death, but its potential prognostic value in acute myeloid leukemia (AML) remains unclear. On the basis of available AML data from TCGA and TARGET databases, a 10-gene signature model was constructed to effectively predict AML prognosis by performing LASSO Cox regression analysis, which showed that patients with a low-risk score had a significantly better prognosis than that of the high-risk group, and receiver operator characteristic (ROC) analysis achieved superior performance in the prognostic model. The model was further well-verified in an external GEO cohort. Multivariable Cox regression analysis showed that, in addition to age, the risk score was an independent poor survival factor for AML patients, and a nomogram model was constructed with high accuracy. Moreover, the high-risk group generally had higher cytolytic activity and increased levels of infiltrating immune cells, including tumor-infiltrating lymphocytes (TILs) and regulatory T cells (Tregs), which could be related to the expression of immune checkpoint genes. Additionally, low-risk AML patients may have a better response from traditional chemotherapeutic drugs. In conclusion, a pyroptosis-related gene signature can independently predict the prognosis of AML patients with sufficient predictive power, and pyroptosis plays an important role in the immune microenvironment of AML, which may be used to develop a new effective therapeutic method for AML in the future.

## 1. Introduction

Acute myeloid leukemia (AML) is a highly heterogeneous group of hematological malignancies derived from the abnormal clonal expansion of myeloid hematopoietic progenitor cells [1]. Although AML is a rare cancer, it remains the second most common type of leukemia [2]. AML mostly occurs in adults, with an average diagnostic age of 68 [2]. The 5-year survival rate for AML patients younger than 20 years old is 60–75% [3], however, the survival rate for older patients is miserable; a few studies showed that for patients over 60, the survival rate is only 3–8% [4,5]. The prognosis of AML varies, and is closely related to the immune system, age, and cytogenetic and molecular abnormalities [6,7]. Despite the improvements in AML diagnosis and therapy over the past several decades, the prognosis of AML patients is still dismal [8]. The risk stratification of AML patients by the European LeukemiaNet (ELN) has been widely applied in current clinical practice, which mainly relies on a combination of pretreatment cytogenetic changes and recurrent gene mutations [9]. Recent technological advancements in the quantitative assessment of measurable residual disease (MRD) and the successful development of novel therapeutic drugs, such as FLT3, BCL2, IDH1, and IDH2 inhibitors, prompted ELN to update the ELN genetic risk classification system in 2022 [9]. However, this system is imprecise since it does not consider other known important prognostic factors, such as the aberrant expression of epigenetic changes, coding and noncoding RNAs, microenvironmental features, and the immune response [10]. Thus, novel effective treatment methods and a more sophisticated prognostic model for AML patients are urgently required to be developed.

Pyroptosis is a gasdermin-mediated inflammatory form of programmed necrotic cell death [11] characterized by pore formation, cell swelling, membrane lysis, DNA fragmentation, and the release of proinflammatory cytokines such as IL-1β and IL-18 [12]. In a canonical caspase-1 dependent pyroptotic pathway, caspase-1 is activated by certain inflammasomes. Then, the active caspase-1 cleaves gasdermin D (GSDMD), which perforates the cell membrane by forming nonselective pores, resulting in cell swelling and lysis [13]. Pro-IL-1β and pro-IL-18 are also cleaved by the activated caspase-1 to secrete IL-18 and IL-1β to the outside of the cell through the pores formed by GSDMD [14]. There are also noncanonical pyroptotic pathways that are mediated by caspase-3/4/5/11 [15]. Numerous studies have shown that pyroptosis-related genes play different roles in various types of cancers [16]. A high expression of GSDMB was associated with a low survival rate and a high metastasis rate in breast cancer [16,17], a high level of GSDMD in lung adenocarcinoma was associated with a poor prognosis, while GSDMD could inhibit tumor proliferation and was related with favorable prognosis in non-smallcell lung cancer [18].

However, the potential prognostic value of pyroptosis-related genes in AML still remains obscure. In the present study, we evaluated the expression level of pyroptosis-related genes in the bone marrow between normal samples and AML samples, and developed a pyroptosis-related risk signature model to predict the prognosis of AML patients, which was further verified with an external database and compared with the current ELN 2022 risk stratification system. Then, the impact of pyroptosis on the immune microenvironment was explored. 

## 2. Materials and Methods

### 2.1. Datasets

The RNA sequencing (RNA-seq, FPKM) data of 70 normal bone marrow samples from the GTEx database, 186 AML patients from the TARGET database, and 136 AML patients from the TCGA database with the corresponding clinical data were downloaded from Xena (https://xenabrowser.net/datapages/ accessed on 1 July 2021), and the related clinical data are shown in the Supplementary Data, Table S1. RNA-seq data with the clinical information of 146 AML patients for the external validation cohort were downloaded from the GEO database (https://www.ncbi.nlm.nih.gov/geo/, ID: GSE147515 accessed on 1 July 2021), and the clinical characteristics of the GEO cohort are summarized in Supplementary Data, Table S2.

### 2.2. Differential Analysis of Pyroptosis-Related Genes

A total of 52 pyroptosis-related genes were obtained from GSEA MSigDB (http://www.gsea-msigdb.org/gsea/msigdb/ accessed on 1 July 2021) and reviews of pyroptosis [12,19,20], which are summarized in Supplementary Data, Table S3. The differential expression of the 52 genes between AML and normal bone marrow tissues were analyzed with the limma package of R software. A protein–protein interaction (PPI) network was built using the STRING website (https://string-db.org/ accessed on 1 July 2021) with a cutoff of 0.9 to assess the relationship among the pyroptosis-related genes, and the final data were visualized using Cytoscape software (https://cytoscape.org/, version 4.0.4 accessed on 1 July 2021).

### 2.3. Consensus Clustering Analysis

For AML patients in the TCGA-TARGET cohort, consensus clustering analysis was performed to identify distinct subgroups on the basis of pyroptosis-related genes by using the ConsensusClusterPlus package of R [21]. The optimal k value was chosen as the number of clusters according to the consensus clustering algorithm.

### 2.4. Prognostic Signature Construction

The clinical information of AML patients from the TCGA and TARGET databases were combined. With the survival data, the prognostic value of pyroptosis-related genes was evaluated by univariate Cox regression analysis, and a forest plot was drawn to present the *p*-value, Hazard Ratio and 95% CI of each statistically significant gene via the “forestplot” R package. A cut-off *p* value  <  0.05 was set to identify prognostic genes for further analysis. The least absolute shrinkage and selection operator (LASSO) Cox regression was used to filter out the overfitting genes and construct a prognostic model with the “glmnet” package. The risk score of each patient was calculated as follows: Risk Score = $\sum_{i=1}^{n}\beta i\;x\;\mathrm{Exp}i$ (n: number of prognostic genes, βi: the coefficient for each gene, Expi: the gene expression level). The AML patients from TCGA-TARGET cohort were classified into high- and low-risk groups according to the median risk score. Principal component analysis (PCA) was performed using the “Rtsne” package. The overall survival between the two groups were compared by using Kaplan–Meier analysis. The predictive power was evaluated by performing time receiver-operating characteristic (ROC) analysis with the “survival”, “survminer” and “timeROC” packages of R. An AML cohort from the GEO database (GSE147515) used as the external validation group. The GEO database was normalized with the “limma” package of R, and the batch effect was removed via the “combat” function in the “sva” package of R. The risk score was calculated by the same formula as used for the TCGA-TARGET cohort. The patients in the GSE147515 cohort were also divided into low- or high-risk subgroups according to the median risk score of the TCGA-TARGET cohort, and these subgroups were then compared to validate the prognostic signature model.

### 2.5. Independent Prognostic Analysis

The clinical variables of the AML patients from the TCGA-TARGET cohort and GEO cohort were merged with their risk scores, respectively. Then univariate and multivariate Cox regression models were used for analyzing the possible independent prognostic value of the risk score. The results of univariate and multivariate Cox regression analyses were presented as forest plots.

### 2.6. Construction and Evaluation of a Nomogram

A nomogram model was constructed to predict the patient’s survival probability by integrating risk scores and other independent clinical factors using the R-package “RMS”. Calibration curves were applied to assess the uniformity between observed and predicted overall survival rates. ROC curves were used to verify the nomogram model’s accuracy.

### 2.7. Drug Sensitivity Evaluation

The half-maximal inhibitory concentration (IC50) of chemotherapeutic drugs were predicted in the high- and low-risk groups of AML patients with the “pRRophetic” [22] package of R.

### 2.8. Functional Enrichment and Immune Microenvironment Analysis

AML patients in the TCGA-TARGET cohorts were stratified into two subgroups, “high-risk” and “low-risk” groups, according to the median risk score. The “limma” R package was utilized to identify the differentially expressed genes (DEGs) between the two groups with the cut-off criteria based on |log2FC|  ≥  1 and FDR  <  0.05. Gene Ontology (GO) and Kyoto Encyclopedia of Genes and Genomes (KEGG) analysis were performed with the “clusterProfiler” package based on the DEGs. The single-sample gene-set enrichment analysis (ssGSEA) program was used to measure the relative abundance infiltrating immune cells and to assess the activity of immune function and pathways with the given immune signature (Supplementary Data, Table S5). Furthermore, the expression level of immune checkpoint genes [23] were compared among different risk groups.

### 2.9. Statistical Analysis

All statistical analysis of the present study was carried out using the R program language (http://www.r-project.org/, version 4.0.4; R Foundation for Statistical Computing, Vienna, Austria accessed on 1 July 2021). For data for more than the two groups, one-way analysis of variance (ANOVA) and the Kruskal-Wallis test were employed for data analysis, respectively. Comparisons between the two groups of continuous data and categorical data were performed using independent T-test and chi-square test, respectively. The Fisher exact method was applied when the smallest expected value is less than five. All *p* values were two-sided and a *p* value < 0.05 was considered to be statistically significant.

## 3. Results

### 3.1. Identification of Differentially Expressed Pyroptosis Genes between Normal and AML Samples

**Figure 1:**
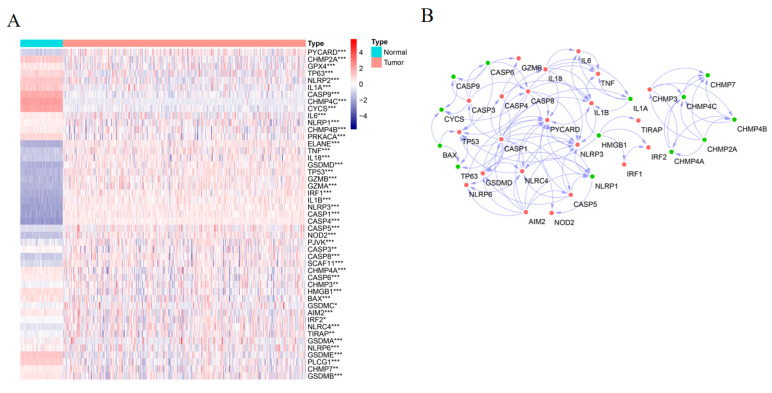
Expressions of the 52 pyroptosis-related genes in AML. (**A**) DEGs Heatmap of the pyroptosis-related genes in the normal (Normal, cyan) and the tumor tissues (Tumor, pink) (purple: low expression level; red: high expression level). *p* values were shown as: * *p* < 0.05; ** *p* < 0.01; *** *p* < 0.001. (**B**) PPI network indicated the interactions of the DEGs (interaction score = 0.9) (red: upregulated; green: downregulated; arrow: interaction).

The 52 pyroptosis-related gene expression levels were compared in 70 normal bone marrow samples from GTEx database, 186 AML samples from TARGET database and 136 AML samples from TCGA database, and a total of 47 differentially expressed genes (DEGs) were identified (all *p* < 0.05, Figure 1A). More specifically, the expression of 19 genes (*CHMP4C*, *CYCS, NLRP2, GSDME, CASP9, PLCG1, IL1A, TP63, CHMP2A, PRKACA, HMGB1, BAX, GSDMB, CHMP4A, GPX4, CHMP4B, CHMP7, NLRP1, CASP6*) were downregulated while the expression of 28 other genes (*CASP3, GSDMA, AIM2, NLRP6, CHMP3, IRF2, TIRAP, SCAF11, PJVK, CASP8, GSDMC, IL6, PYCARD, IL18, GSDMD, NLRC4, IRF1, TP53, TNF, NOD2, CASP5, CASP4, GZMB, CASP1, NLRP3, IL1B, GZMA, ELANE*) were upregulated in AML compared with normal samples. To further explore the interactions among these pyroptosis-related genes, a protein-protein interaction (PPI) analysis was performed with the minimum required interaction score setting at 0.9 (the highest confidence), and the results were processed by using the software Cytoscape and shown in Figure 1B.

### 3.2. AML Clustering Based on the DEGs

**Figure 2:**
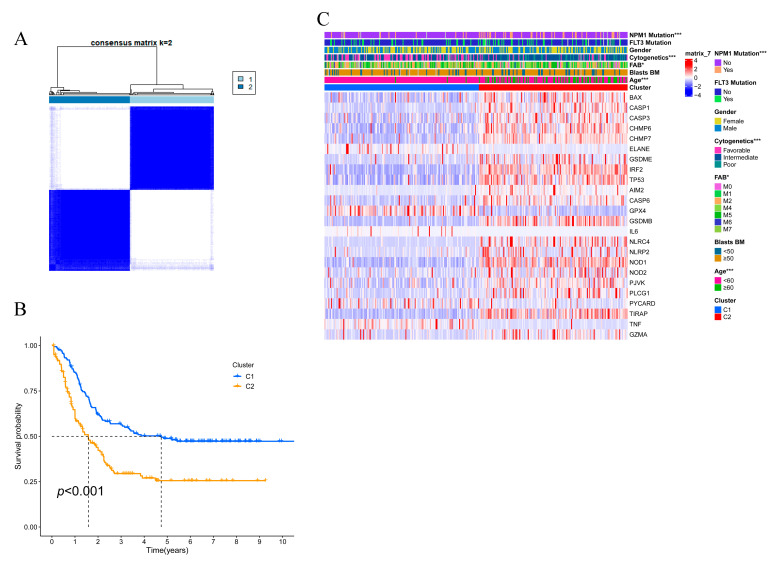
AML clustering based on the differentially expressed pyroptosis-related genes. (**A**) All AML patients with available survival data from the TCGA-TARGET cohort were divided into two clusters according to the consensus clustering matrix (k = 2). (**B**) Kaplan–Meier overall survival curves of the two clusters. C1, Cluster1; C2, Cluster2. (**C**) Heatmap and clinical features of the two clusters. * *p* < 0.05; *** *p* < 0.001.

To investigate the connections between the expression of the 47 pyroptosis-related DEGs and AML subtypes, a consensus clustering analysis was performed in the TCGA-TARGET AML cohorts with available survival information (a total of 322 samples). The clustering variable k = 2 was identified with optimal clustering stability by increasing k from 2 to 9. And all the 322 AML patients were well divided into two clusters based on the 47 DEGs (Figure 2A). The overall survival (OS) was compared between the two clusters, Cluster1 (C1) group showed significantly better prognosis than the Cluster2 (C2) group (*p* < 0.001, Figure 2B). The gene expression profile and the clinical characteristics including gender, cytogenetic risk (favorable, intermediate, poor), age (≤60 or >60 years), blasts of BM (bone marrow) (<50%, ≥50%), the status of mutated genes (NPM1, FLT3) and the cluster group were compared in a heatmap, and it was found that in the Cluster 1 group, there are significantly more cases with the clinical features of younger ages (≤60 years), favorable cytogenetics, without mutation of NPM1 and FLT3, and more blasts in BM (≥50%) (Figure 2C). 

### 3.3. Construction of a Prognostic Model in the AML Cohort

**Figure 3:**
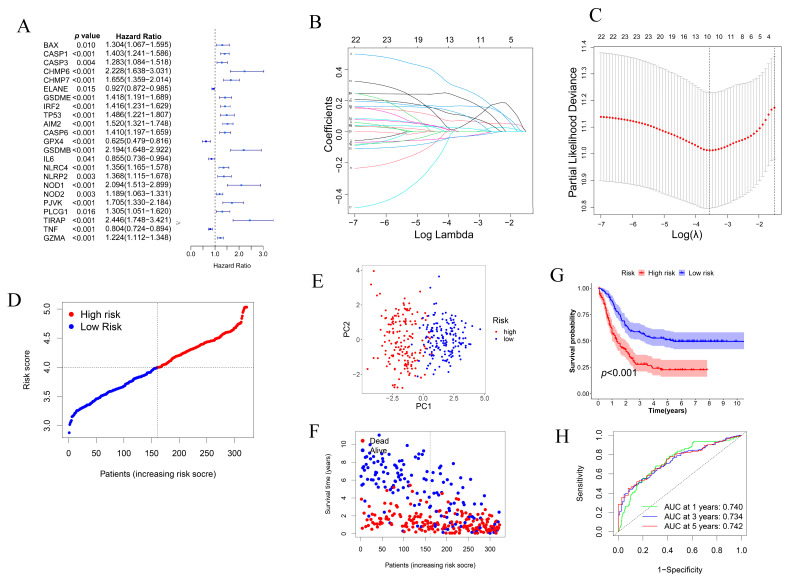
Establishment of a risk signature model of AML patients in the TCGA-TARGET cohort. (**A**) Univariate cox regression analysis of OS for the statistically different expressed pyroptosis-related gene, the results were shown as a forest plot, and 23 genes with *p* < 0.05. (**B**) LASSO -COX regression of the 23 survival-related genes. (**C**) Cross-validation for the tuning parameter (λ) selection in the LASSO regression. (**D**) Distribution of AML patients based on their risk scores. (**E**) PCA plot for AML patients based on the risk score. (**F**) Distribution of survival status of AML patients. (**G**) Kaplan-Meier survival curve for the high-risk and low-risk groups. (**H**) The time-dependent ROC curves showed the predictive efficiency of the risk signature model.

Univariate Cox regression analysis was performed to identify survival-related genes. Finally, 23 genes were found to be statistically significant and were retained for further analysis (*p* < 0.05, Figure 3A). Among them, 19 genes (*BAX, CASP1, CASP3, CHMP6, CHMP7, GSDME, IRF2, TP53, AIM2, CASP6, GSDMB, NLRC4, NLRP2, NOD1, NOD2, PJVK, PLCG1, TIRAP* and *GZMA*) with HR > 1 were associated with increased risk, while the other 4 genes (*ELANE, GPX4, IL6* and *TNF*) with HR < 1 were protective genes (Figure 3A). To prevent overfitting, LASSO-Cox regression analysis was performed for the 23 genes, and a 10-gene signature was established with the optimum λ value (Figure 3B,C). The risk score was calculated as follows: Risk Score = (0.070 × CASP1 exp.) + (0.276 × CHMP6 exp.) + (−0.032 × ELANE exp.) + (0.104 × GSDME exp.) + (0.164 × AIM2 exp.) + (0.035 × GSDMB exp.) + (0.110 × NLRP2 exp.) + (0.213 × PJVK exp.) + (−0.032 × TNF exp.) + (0.023 × GZMA exp.). All the 322 AML patients were then equally divided into low- and high-risk subgroups according to the median score calculated by the risk score formula (Figure 3D). PCA analysis showed that patients with different risks were well distributed into two clusters (Figure 3E). With the increase of risk score, more deaths were found (Figure 3F). The low-risk group had a significantly better overall survival than the high-risk group (*p* < 0.001, Figure 3G). To assess the specificity and sensitivity of the prognostic model, ROC analysis was performed, and we found that the area under the ROC curve (AUC) was 0.740 for 1-year, 0.734 for 3-year, and 0.742 for 5-year survival, respectively (Figure 3H).

### 3.4. External Validation of the Prognostic Model

**Figure 4:**
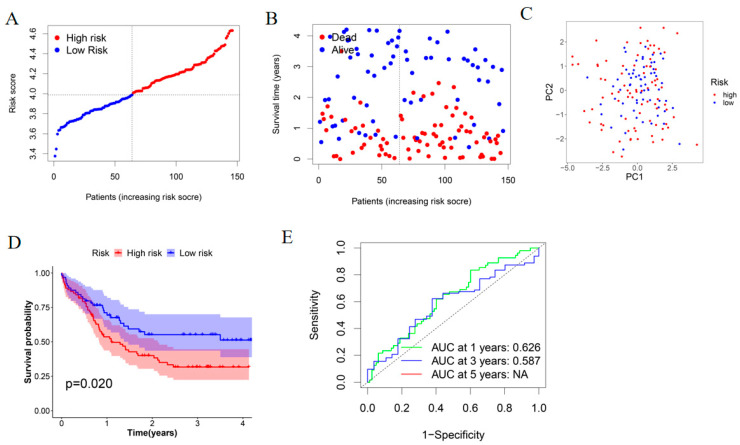
Verification of the prognostic risk model in the GEO cohort. (**A**) Distribution of AML patients in the GEO cohort based on their risk score. (**B**) The survival status distribution of each patient in the GEO cohort. (**C**) PCA plot for AML patients in the GEO cohort. (**D**) Kaplan-Meier curve of high-risk and low-risk groups. (**E**) Time-dependent ROC curves for AML patients in the GEO cohort.

A total of 146 AML patients with survival data from a GEO cohort (GSE147515) were employed as the external validation set. According to the median risk score calculated in the TCGA-TARGET AML cohort, 82 patients in the GEO cohort were allotted into the high-risk group, while the other 64 patients were allotted into the low-risk group (Figure 4A). Consistent with the TCGA-TARGET cohort, with increasing risk score, more dead patients were found (Figure 4B). PCA analysis demonstrated satisfactory separation between the two groups (Figure 4C). Kaplan-Meier survival analysis also showed significant differences between the two groups (*p* = 0.020, Figure 4D). Time-dependent ROC analysis of the GEO cohort indicated that the prognostic model had a good predictive efficacy (AUC = 0.626 for 1-year, 0.587 for 3-year) (Figure 4E).

Moreover, to compare our model with the traditional ELN risk classification system that is widely used in the clinic [9], AML patients were stratified into three risk groups (favorable, intermediate, and poor) according to their cytogenetics and molecular profiles. After re-stratification of AML patients (Figure S1A,C), there were no significant differences of overall survival after applying the ELN classification for the TCGA-TARGET cohort (Figure S1B). For the GEO cohort, there were only significant differences between the intermediate and favorable group (*p* = 0.018, Figure S1D). Taken together, the pyroptosis risk model could more accurately define AML patients’ prognosis than the ELN risk stratification system.

### 3.5. Evaluation of the Independent Prognostic Value of the Risk Model

**Figure 5:**
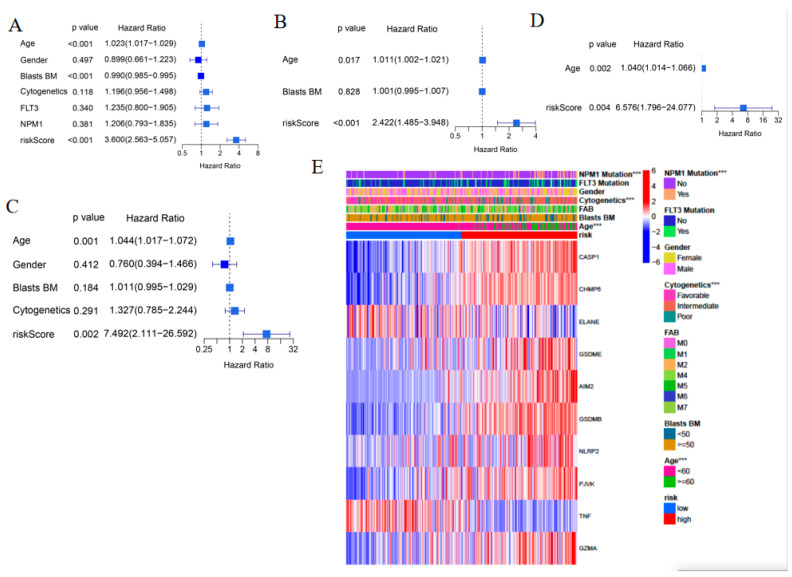
Univariate and multivariate Cox regression analyses of the risk score and clinical features. (**A**,**C**) Univariate analysis of the TCGA-TARGET cohort (**A**) and GEO cohort (**C**) (Cytogenetics: favorable, intermediate, poor; Blasts BM: blasts percentage in the bone marrow). (**B**,**D**) Multivariate analysis of the TCGA-TARGET cohort (**C**) and the GEO cohort (**D**). (**E**) Heatmap (blue: low expression; red: high expression) of clinical characteristics in the TCGA-TARGET cohort between the high-risk and low-risk groups (*** *p* < 0.001).

To determine the independent prognostic value of the risk score, univariate and multivariable Cox regression analyses were performed. The univariate Cox regression analysis showed that in addition to age, the risk score was an independent poor survival factor in both the TCGA-TARGET and GEO cohorts (HR = 3.600, 95% CI: 2.563–5.057 and HR: 7.492, 95% CI: 2.111–26.592, respectively. Figure 5A,C). Multivariate analysis further indicated that the risk score was a poor survival prognostic factor (TCGA-TARGET: HR = 2.422, 95% CI: 1.485–3.948; GEO: HR = 6.576, 95% CI: 1.796–24.077, Figure 5B,D). Multivariate analysis also demonstrated that age was a poor survival prognostic factor (*p* = 0.002). Moreover, a heatmap of the clinical characteristics of the TCGA-TARGET cohort showed there are significant different distribution of age, cytogenetics, and mutation of *NPM1* between the low-risk and high-risk subgroups (Figure 5E, *p* < 0.05). 

### 3.6. Establishment and Evaluation of a Nomogram Model

**Figure 6:**
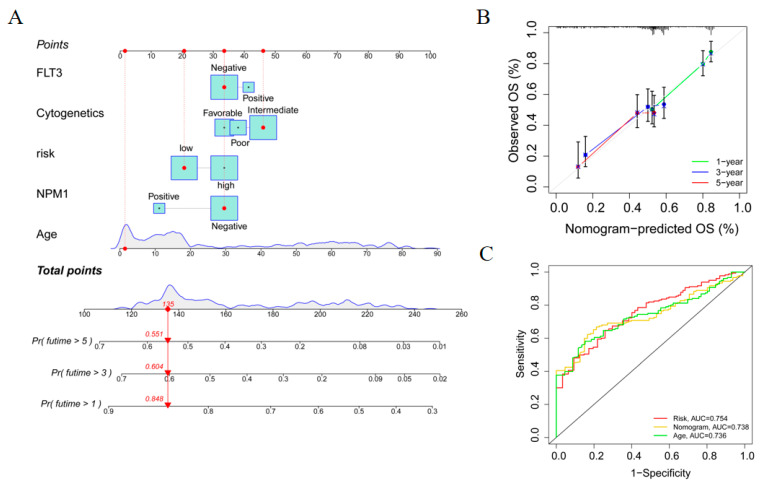
Construction and evaluation of the nomogram of AML patients in the TCGA-TARGET cohort. (**A**) The nomogram for predicting the probability of patient survival with risk groups and clinical features. (**B**) The calibration diagram of the nomogram for the observed overall survival (OS) probability and predicted OS at 1-year, 3-year, and 5-year intervals. (**C**) ROC curve analyses of the nomogram.

A nomogram was further constructed to predict the patient’s 1-, 3-, and 5-year survival probability by integrating risk factors and other independent clinical variables (Figure 6A). The calibration chart of the nomogram showed that the predicted and observed survival probability matched well at 1-, 3-, and 5-year intervals (Figure 6B). Moreover, the ROC curve showed that the 1-year AUC of the nomogram was 0.738, similar to other clinical features (risk, AUC = 0.754; age, AUC = 0.736; Figure 6C). These results indicate that the nomogram model had high accuracy, asserting its practicability in future clinical application for predicting the prognosis of AML patients.

### 3.7. Difference of Immune Microenvironment between Subgroups

**Figure 7:**
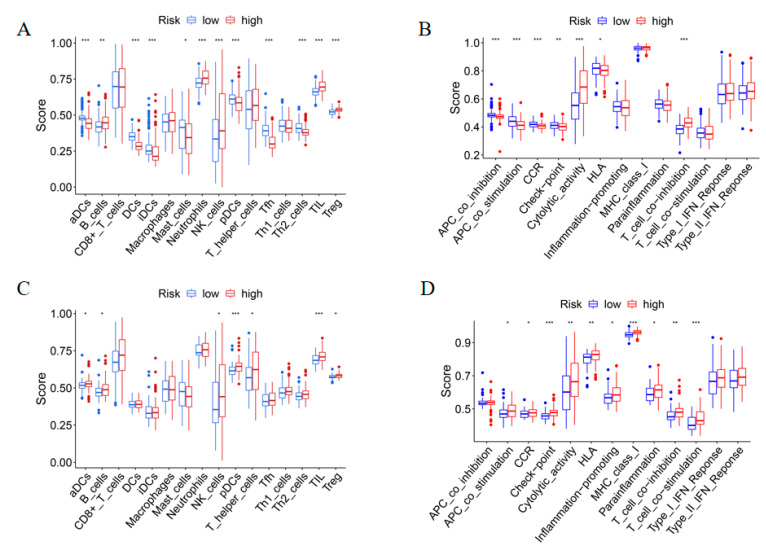
Comparison of the ssGSEA scores of immune cells and immune pathways for two subgroups in both the TCGA-TARGET and GEO cohorts. (**A**,**B**) Enrichment scores of 16 types of immune cells (**A**) and 13 immune-related pathways (**B**) between low- (green box) and high-risk (red box) group in the TCGA cohort. (**C**,**D**) Enrichment scores of 16 types of immune cells (**C**) and 13 immune-related pathways (**D**) between low- (green box) and high-risk (red box) group in the GEO cohort. * *p* < 0.05; ** *p* < 0.01; *** *p* < 0.001.

**Figure 8:**
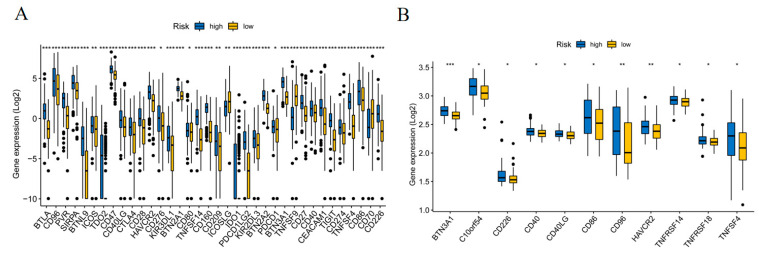
Expression comparison of immune checkpoint genes between different risk groups in the TCGA-TARGET cohort (**A**) and GEO cohort (**B**). * *p* < 0.05, ** *p* < 0.01, *** *p* < 0.001, and **** *p* < 0.0001.

Based on the results of functional analyses, single-sample gene set enrichment analysis (ssGSEA) was performed to compare the immune microenvironment between the low and high-risk groups in both the TCGA-TARGET and GEO cohorts with the immune gene set consisting of 16 types of immune cells and the activity of 13 immune-related pathways. In both cohorts, the high-risk subgroup generally had higher levels of infiltration of immune cells, especially B cells, natural killer (NK) cells, T helper (Th) cells, tumor-infiltrating lymphocytes (TILs), and regulatory T (Treg) cells, than the low-risk subgroup (Figure 7A,C). In addition, it was found that cytolytic activity and T cell co-inhibition were consistently upregulated in the high-risk subgroup compared to the low-risk group (Figure 7B,D). As these results indicated that increasing the risk score of AML cells showed higher inhibitory immune microenvironment, the expression levels of immune checkpoints (ICPs) were assessed in the two risk subgroups. A total of 59 ICPs-related genes were compared between the high-risk and the low-risk group in the TCGA-TARGET and GEO cohorts. The high-risk group had significantly higher expression of 32 ICPs including *CD28, CTLA4, TIGIT, PDCD1*(*PD-1*) and *PDCD1LG2* (*PD-L2*) in the TCGA-TARGET cohort (Figure 8A, Student’s *t*-test, * *p*  <  0.05; ** *p*  <  0.01; *** *p*  <  0.01; **** *p*  <  0.001). In the GEO cohort, the high-risk group had distinctly higher expression of 11 ICPs including *C10orf54, CD40, CD86, TNFRSF14, TNFRSF14* and *TNFSF4* (Figure 8B, Student’s *t*-test, * *p*  <  0.05; ** *p*  <  0.01; *** *p*  <  0.01; **** *p*  <  0.001). The data suggests that the elevated inhibiting immunologic activity in the high-risk group may be related with the expression of ICPs.

### 3.8. Drug Response Analysis of High and Low-Risk Patients to Chemotherapy

**Figure 9:**
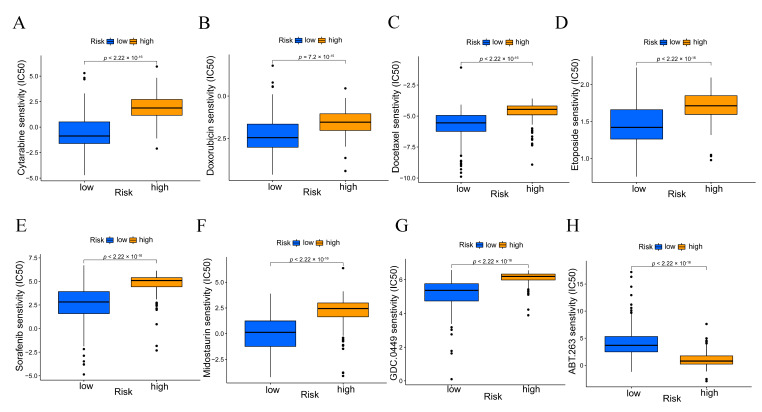
The correlation between different risk groups and drug sensitivity in AML patients. (**A**) Cytarabine; (**B**) Doxorubicin; (**C**) Docetaxel; (**D**) Etoposide; (**E**) Sorafenib; (**F**) Midostaurin; (**G**) GDC-0449; (**H**) ABT-263. IC50, half maximal inhibitory concentration, in nM.

The IC50 of four commonly used chemotherapeutic agents (cytarabine, doxorubicin, docetaxel, and etoposide) and four inhibitors (sorafenib, midostaurin, GDC-0449, and ABT-263) in high- and low-risk AML patients were predicted by using the pRRophetic algorithm. It was found that all the four chemotherapeutic drugs had remarkably higher IC50 in the high-risk patients (Figure 9A–D). The two inhibitors sorafenib and midostaurin which are beneficial for AML patients with the *FLT3* mutations, both showed significantly higher IC50 in the high-risk group (Figure 9E,F), and the hedgehog inhibitor GDC-0449 (Vismodegib) also exhibited remarkably elevated IC50 in the high-risk group (Figure 9G), while the BCL-2 inhibitor ABT-263 showed significantly lower IC50 in the high-risk group (Figure 9H). Moreover, the mRNA expression level of *PTCH1, SMO, GLI2* and *BCL2* were significantly elevated in the high-risk group (Figure S2). The data suggests that the patients with low-risk scores had a more sensitive response to the traditional chemotherapy commonly applicated in AML therapy in a clinical setting. Low-risk patients may also be more sensitive to both sorafenib and midostaurin which are used for AML patients with *FLT3* mutations, and to the hedgehog inhibitor vismodegib, but high-risk patients could be more sensitive to the BCL-2 inhibitor ABT-263.

### 3.9. Functional Enrichment Analyses of the Risk Model

**Figure 10:**
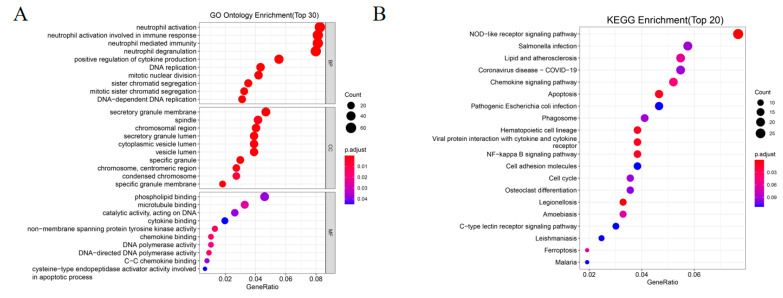
Functional enrichment analysis based on the DEGs between high-risk and low-risk groups in the TCGA-TARGET cohort. (**A**) Bubble graph for GO enrichment (BP: biological process, CC: cellular component, MF: molecular function; *p*.adjust: the adjusted *p*-value). (**B**) Bubble graph for KEGG pathways.

To further clarify the differences in the biological functions and pathways between the subgroups categorized by the risk model, enrichment analyses were performed based on the DEGs between the high-risk and the low-risk group in the TCGA-TARGET cohort by applying the criteria FDR < 0.05 and |log2FC| ≥ 1. A total of 853 DEGs were identified at first (Supplementary Data, Table S4). Among them, 66 genes were upregulated in the high-risk group, while the other 49 genes were downregulated (Supplementary Data, Table S4). Gene ontology (GO) enrichment analysis and Kyoto Encyclopaedia of Genes and Genomes (KEGG) pathway analysis were then performed. The results suggest that the DEGs were mainly enriched in the innate immune response, cytokine production chemokine signaling pathways, cell cycle, DNA replication and apoptosis (Figure 10A,B), which may provide indications for the poor survival and resistance to chemotherapeutic drugs in the high-risk subgroup.

## 4. Discussion

Pyroptosis is a newly defined gasdermin-mediated inflammatory form of programmed cell death, which exerts a dual function in cancer progression [11]. On the one hand, pyroptosis can activate various signaling pathways and release diverse inflammatory factors that are tightly linked with tumorigenesis and drug resistance [13,16]. On the other hand, pyroptosis can promote cancer cell death making it a potential therapeutic target for cancer therapy [24]. However, until now, the significance of pyroptosis in AML has not been fully studied.

Many studies have shown that pyroptosis via the caspase-1 dependent canonical pathway is mostly triggered by inflammasomes including NLRP1, NLRP3, NLRC4 and AIM2 [11,25]. These inflammasomes can activate caspase-1 to cleave GSDMD that will induce pyroptosis [26]. Caspase-1 will also cleave Pro-IL-1β and pro-IL-18 that will be secreted to outside of the cell through the membrane pores formed by GSDMD as IL-18 and IL-1β [27]. There are also non-canonical pyroptotic pathways that are mediated by GSDMA, GSDMB, GSDMC, GSDME, and GZMB. The cell membrane disintegrity induced by pyroptosis can cause influx of Ca^2+^, which will recruit ESCRT-III complex to repair the damaged plasma membranes [28]. Several major ESCRT-III components have been discovered so far, including charged multivesicular body protein 1A (CHMP1A), CHMP1B, CHMP2A, CHMP2B, CHMP3, CHMP4A, CHMP4B, CHMP4C, CHMP5, CHMP6, and CHMP7 [29]. In this study, we found that most of the pyroptosis-related genes were differentially expressed in AML and normal controls, and the level of the membrane repair genes such as *CHMP2A, CHMP4A, CHMP4B, CHMP4C, CHMP7*, were downregulated while the level of canonical and non-canonical pyroptosis genes such as *GSDMD, NLRP3, NLRC4, AIM2, GSDMA, GSDMB, GSDMC, GSDME, GZMB, IL18* and *IL1B* were upregulated in AML compared with normal samples. Our data indicated that pyroptosis was activated while the membrane repair process was impaired in AML. Some research demonstrated that pyroptosis-related genes play different roles in various types of cancers [16]. It was found that a high expression of GSDMB was associated with a low survival rate and a high metastasis rate in breast cancer [16,17], and a high level of GSDMD in lung adenocarcinoma was associated with a poor prognosis, while GSDMD can inhibit tumor proliferation and was related with favorable prognosis in non-small cell lung cancer [18]. In this study, we found that the pyroptosis genes including *GSDMB, GSDME, NLRC4, NLRP2* and *GZMA* were hazardous genes in AML, and the genes of proinflammatory cytokines such as *IL6* and *TNF* were found to be protective genes which maybe related with tumor microenvironment.

Some recent studies have identified specific pyroptosis-related gene signatures in some cancers: a pyroptosis-related signature consisting of four genes was constructed to predict the prognosis of thyroid cancer [30]; a 10 pyroptosis-related gene signature was built to predict the prognosis of glioma [31]; a novel pyroptosis-related gene signature was defined to predict the prognosis of ovarian cancer [32]. However, the potential prognostic value of pyroptosis-related gene signature in AML has not yet been studied. In our study, we built a 10-gene signature model that can independently predict the prognosis for AML patients, which showed that the prognosis of AML deteriorates with increasing risk scores. ROC analysis presented a large area under the ROC curve (AUC) indicating the superior performance of our risk signature model. Univariate and multivariable Cox regression analyses showed that in addition to age, the risk score was an independent survival factor for AML patients in both the TCGA-TARGET and GEO cohorts. The ELN risk stratification is a widely used scheme for AML patients [9], but we found that the pyroptosis risk model in this study seemed to more accurately define AML patients’ prognosis than the ELN risk stratification system. However, this was just a preliminary and limited finding, further research is warranted to make a better risk stratification system for the AML patients. Our data suggests that the risk score feature may be considered as an independent and crucial prognostic predictor for AML. In addition, to prove the possibility of clinical application of the risk signature, a nomogram model was established with high accuracy in predicting the patient’s overall survival probability, which could help physicians make optimal therapy plans for AML patients accordingly.

In the present study, in both the TCGA-TARGET and GEO AML cohorts, the high-risk group generally presented increased levels of infiltration of immune cells such as B cells, natural killer (NK) cells, T helper (Th) cells, tumor-infiltrating lymphocytes (TILs) and regulatory T (Treg) cells, and cytolytic activity and T cell co-inhibition were consistently elevated in the high-risk group as well. The data indicates that the bone marrow of the high-risk group of AML patients were infiltrated with immune cells, but their immune function was suppressed by Tregs, suggesting the important role of pyroptosis in the tumor microenvironment (TME) landscapes of AML. Lots of convincing data have already demonstrated that AML cells can escape immune surveillance by adapting themselves to the bone marrow TME through altering the immune cell function as a consequence of a dysregulated cytokine network directly mediated by AML cells [33]. Some comprehensive studies show that AML cells promote T-cell exhaustion by driving the expansion of Tregs [34]. It has also been reported that there was comparatively high level of Tregs in the bone marrow of AML patients, and an elevated number of Tregs in AML is associated with poor prognosis [35,36,37]. Additional studies revealed that the enrichment of Tregs in the TME of AML was related to the secretion of immunoinhibitory factors, such as transforming growth factor-β (TGF-β), IL-10 and IL-35 by AML blasts [38]. Thus, based on these findings, the poor survival outcome of the high-risk group of AML patients may be related to immune suppression in the TME of bone marrow caused by immunosuppressive effector cells, such as Tregs. ICPs are normally regulatory molecules which can maintain immune homeostasis in a healthy body; however, lots of studies found that they are overexpressed in various tumors to suppress the anti-cancer immune response in the TME [39]. In this study, we found that the high-risk group showed higher expression level of ICPs including *CD28, CTLA4, TIGIT, PDCD1*(*PD-1*) and *PDCD1LG2*(*PD-L2*), which may contribute to the inhibiting immunologic microenvironment in AML. In addition, the high-risk group significantly corresponded to enrichment pathways associated with immune activation including the NOD-like receptor signaling pathway, neutrophil activation, cytokine binding, cytokine receptor interaction and the chemokine signaling pathway. However, how pyroptosis promotes the differentiation of immunosuppressive microenvironment including Tregs is currently unclear.

Finally, in this study, we found that the low-risk AML patients may benefit more from traditional chemotherapeutic drugs (cytarabine, doxorubicin, docetaxel, and etoposide); low-risk patients may also perform better to both sorafenib and midostaurin that are used for AML patients with *FLT3* mutations and to the hedgehog inhibitor GDC-0449 (Vismodegib), while high-risk patients will have better responses to the BCL-2 inhibitor ABT-263. Moreover, the main targets of vismodegib *PTCH1, SMO* and their downstream target gene *GLI2* were all found to be upregulated in the high-risk group in this study. Increased expression of *PTCH1* and/or *SMO* will lead to the upregulation of hedgehog target genes including *GLI2*. A study showed that the upregulation of *GLI2* was implicated in resistance to vismodegib in medulloblastoma [40], which is consistent with our data. In addition, our data demonstrated that BCL2 was also significantly elevated in the high-risk group. It was found that BCL-2 was overexpressed in 87% *de novo* AML and in nearly 100% of relapsed AML patients [41]. Another study showed that Bcl-2 expression correlated very well with the sensitivity of the BCL-2 inhibitor venetoclax in various diffuse large B-cell lymphoma (DLBCL) cell lines [42]. Thus, the elevated sensitivity to the BCL-2 inhibitor in the high-risk group may be related to the higher expression level of BCL2 in this group. Cytarabine and doxorubicin-based chemotherapies are the standard regimen for the treatment of AML [43,44], however, the mechanism of heterogeneity in drug response is unknown. The results of enrichment analysis in this study indicate that the high-risk group was significantly enriched with cell cycle, DNA replication pathways, inflammatory response, and the NFκB signaling pathway. Interestingly, inflammatory response, the NFκB signaling pathway and cell cycle pathways such as E2F targets have already been found to be correlated with the oncogenesis and progression of AML [45,46,47,48]. Thus, those enriched pathways in the high-risk group may be associated with their resistance to chemotherapeutic drugs. This study revealed the different sensitivity of low- and high-risk AML patients to the four clinically used chemotherapeutic drugs including cytarabine and doxorubicin, which will provide more rational choices of chemotherapy for AML patients with a low-risk signature of pyroptosis.

In conclusion, we constructed a risk signature model consisting of 10 pyroptosis-related genes possessing sufficient predictive power to independently predict the prognosis of AML patients, which seemed more precise than the ELN 2022 risk stratification system. Compared to the low-risk group, the high-risk group had an elevated inhibitory immune microenvironment which may be associated with higher expression of ICPs. Moreover, the risk signature can predict the sensitivity of AML patients to four commonly used chemotherapeutic agents (cytarabine, doxorubicin, docetaxel, and etoposide) as well as four small-molecular inhibitors (sorafenib, midostaurin, vismodegib and ABT-263) and will provide some clues for new clinical applications in AML patients. Our study also provides a novel perspective of a pyroptosis-related signature for predicting the prognosis of AML and suggests that pyroptosis regulation in the TME of AML may be an effective therapeutic strategy for the future treatment of AML. Nonetheless, further research with real-world data is still required to consolidate our findings.

## Data Availability

The datasets analyzed during this study are available at TCGA, TARGET, UCSC Xena and GEO database (https://portal.gdc.cancer.gov/, accessed on 1 July 2021, https://ocg.cancer.gov/programs/target/, accessed on 1 July 2021 https://xenabrowser.net/ accessed on 1 July 2021 and https://www.ncbi.nlm.nih.gov/geo/; GSE147515 accessed on 1 July 2021).

## Supplementary Materials

The following supporting information can be downloaded at: https://www.mdpi.com/article/10.3390/genes13122281/s1, Figure S1: ELN2022 risk stratification system; Figure S2: Expression comparison of PTCH1, SMO, GLI2 and BCL2 between low and high risk groups in the TCGA-TARGET cohort; Table S1: Clinical characteristics of patients in the TCGA-TARGET cohort; Table S2: Clinical characteristics of patients in the GEO cohort; Table S3.xlsx: Supplementary Data, Table S3; Table S4.xlsx: Supplementary Data, Table S4; Table S5.xlsx: Supplementary Data, Table S5.

## Abbreviations

AML: Acute myeloid leukemia; AUC, Area under the curve; BM, Bone Marrow; FLT3, fms related tyrosine kinase 3; GTEx, Genotype-Tissue Expression; GEO, Gene Expression Omnibus; GSEA, Gene set enrichment analysis; GO, Gene ontology; HSC, Hematopoietic stem cell; ICPs, immune checkpoints; KEGG, Kyoto encyclopedia of genes and genomes; LASSO, Least absolute shrinkage and selection operator; NPM1, nucleophosmin 1; ROC, Receiver operating characteristic; ssGSEA, single-sample gene set enrichment analysis; TARGET, Therapeutically Applicable Research to Generate Effective Treatments; TCGA, The Cancer Genome Atlas; TME, Tumor microenvironment; UCSC, University of California Santa Cruz.
