# Supplementary material for: A Novel Defined Pyroptosis-Related Gene Signature for the Prognosis of Acute Myeloid Leukemia
Source: Genes (Basel). 2022 Dec 3;13(12):2281. doi: 10.3390/genes13122281 (PMC9778227; doi:10.3390/genes13122281)
Supplement: Supplementary file 1 [file genes-13-02281-s001.zip › Table S1.pdf]

Table S1. Clinical characteristics of patients in the TCGA-TARGET cohort

Characteristics	Overall (N=322)
**Age (Years)**	
Mean (SD)	29.0 (25.3)
Median [Min, Max]	17.0 [1.00, 88.0]
**Gender, n (%)**	
Female	154 (47.8%)
Male	168 (52.2%)
**Blasts BM, (%)**	
Mean (SD)	69.4 (20.5)
Median [Min, Max]	72.0 [14.0, 100]
NA, n (%)	6 (1.9%)
**Cytogenetics, n (%)**	
Favorable	87 (27.0%)
Intermediate	179 (55.6%)
Poor	46 (14.3%)
NA	10 (3.1%)
**FAB, n (%)**	
M0	20 (6.2%)
M1	56 (17.4%)
M2	76 (23.6%)
M4	80 (24.8%)
M5	50 (15.5%)
M6	5 (1.6%)
M7	11 (3.4%)
NA	24 (7.5%)
**FLT3 Mutation*, n (%)**	
No	248 (77.0%)
Yes	67 (20.8%)
NA	7 (2.2%)
**NPM1 Mutation, n (%)**	

Characteristics	Overall (N=322)
No	268 (83.2%)
Yes	46 (14.3%)
NA	8 (2.5%)

*FLT3 mutation includes FLT3-ITD and FLT3-PM.

NA, not available; BM, bone marrow.
