# Supplementary material for: A Novel Defined Pyroptosis-Related Gene Signature for the Prognosis of Acute Myeloid Leukemia
Source: Genes (Basel). 2022 Dec 3;13(12):2281. doi: 10.3390/genes13122281 (PMC9778227; doi:10.3390/genes13122281)
Supplement: Supplementary file 1 [file genes-13-02281-s001.zip › Table S2.pdf]

Table S2. Clinical characteristics of patients in the GSE147515 cohort

Characteristics	Overall (N=146)
**Age (Years)**	
Mean (SD)	57.0 (14.5)
Median [Min, Max]	60.0 [18.0, 85.0]
**Gender, n (%)**	
Female	40 (27.4%)
Male	33 (22.6%)
NA	73 (50.0%)
**Blasts BM (%)**	
Mean (SD)	68.1 (19.1)
Median [Min, Max]	70.0 [33.0, 99.0]
NA, n (%)	73 (50.0%)
**Cytogenetics, n (%)**	
Good	14 (9.6%)
Intermediate	115 (78.8%)
Poor	17 (11.6%)
**FAB, n (%)**	
M0	8 (5.5%)
M1	37 (25.3%)
M2	51 (34.9%)
M3	5 (3.4%)
M4	24 (16.4%)
M5	17 (11.6%)
M6	3 (2.1%)
M7	1 (0.7%)

NA, not available; BM, bone marrow.
