# Supplementary figures and images for: A Novel Defined Pyroptosis-Related Gene Signature for the Prognosis of Acute Myeloid Leukemia
Source: Genes (Basel). 2022 Dec 3;13(12):2281. doi: 10.3390/genes13122281 (PMC9778227; doi:10.3390/genes13122281)

## Slide 1
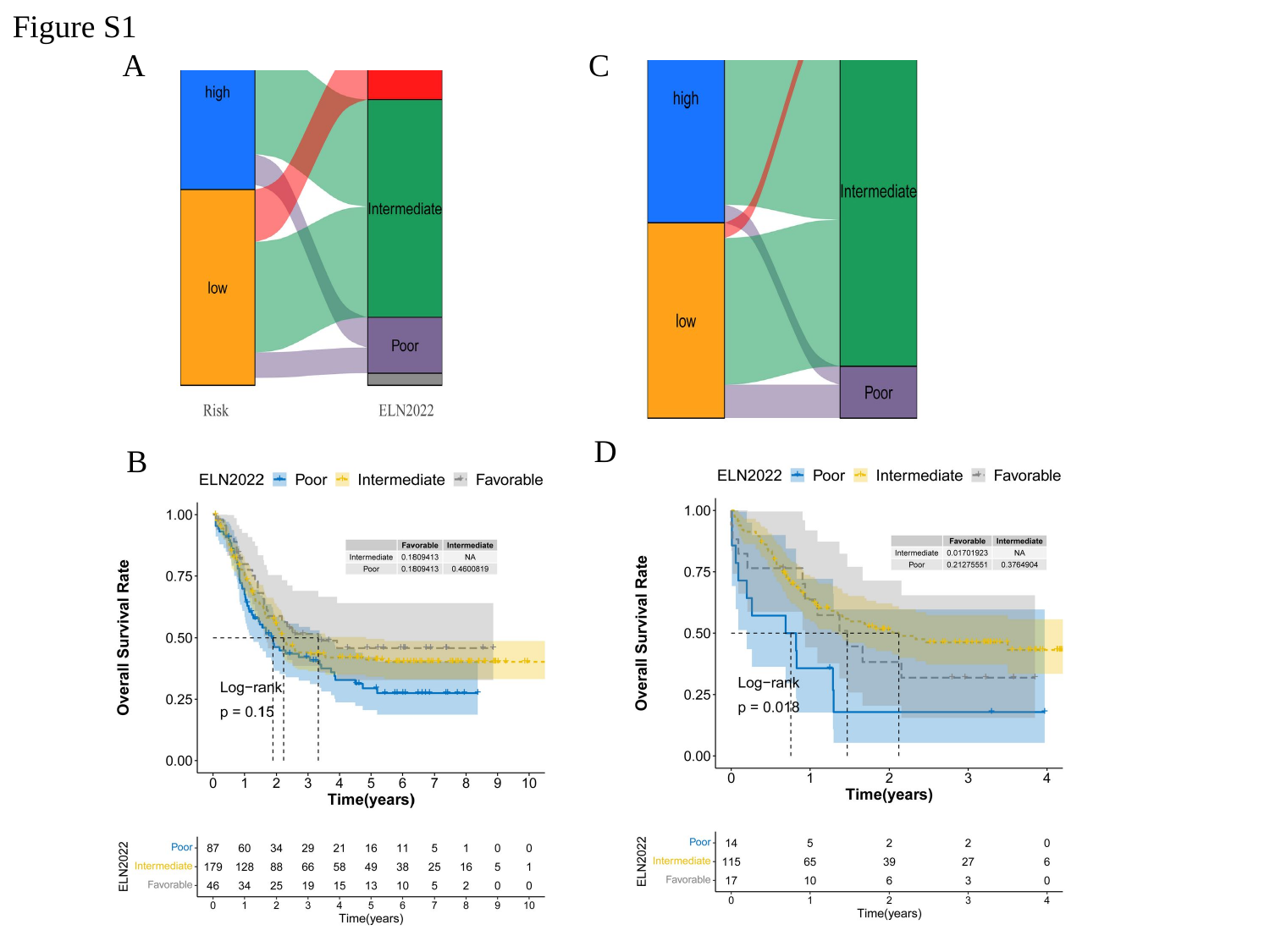

Figure S1
A
C
D
B

## Slide 2
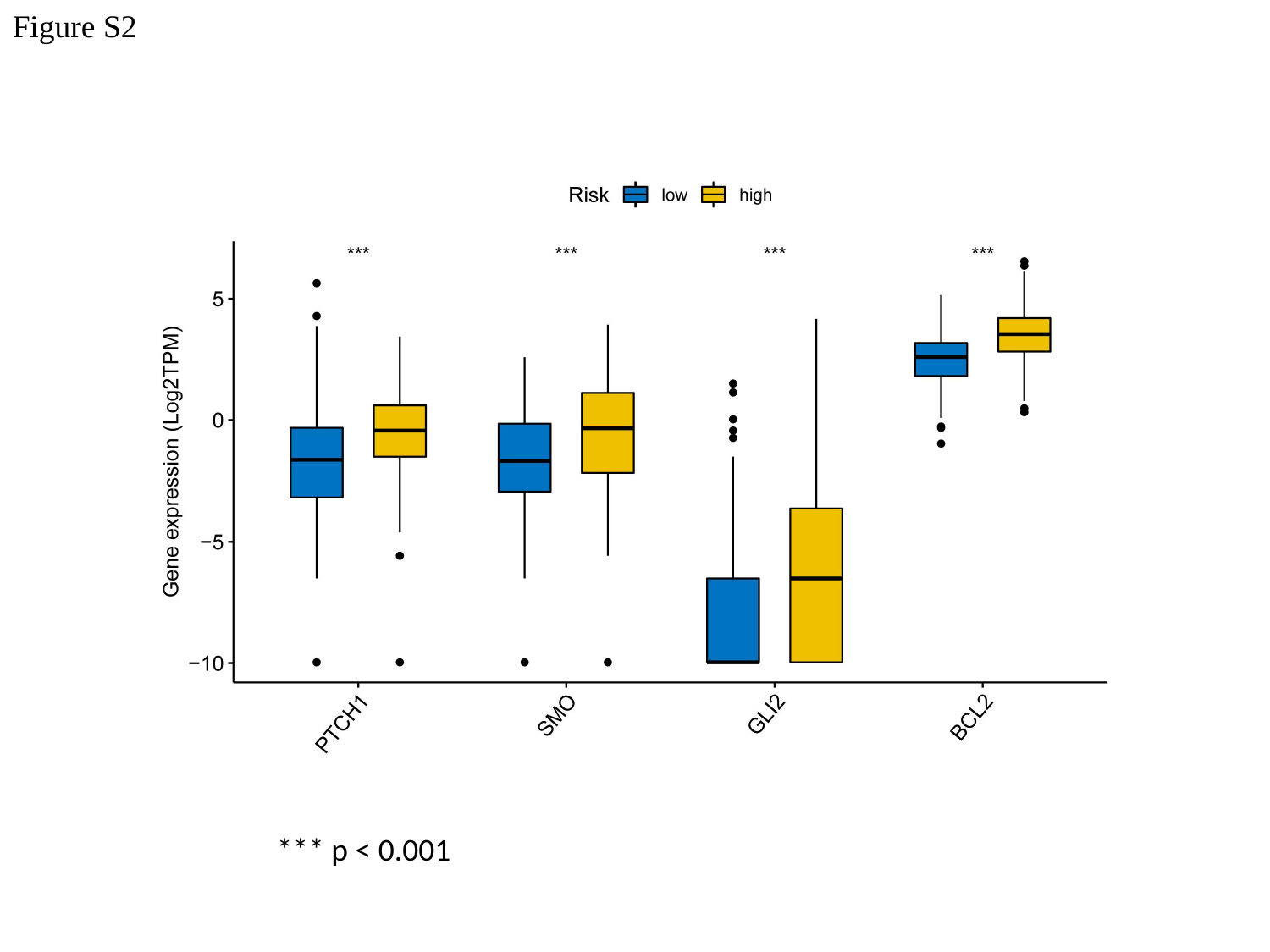

Figure S2
*** p < 0.001

Supplement: Supplementary file 1 [file genes-13-02281-s001.zip › Supplementary Figures.pptx]
